# Test–Retest Reliability of Virtual Reality Devices in Quantifying for Relative Afferent Pupillary Defect

**DOI:** 10.1167/tvst.12.6.2

**Published:** 2023-06-06

**Authors:** Prithul Sarker, Nasif Zaman, Joshua Ong, Phani Paladugu, Molly Aldred, Ethan Waisberg, Andrew G. Lee, Alireza Tavakkoli

**Affiliations:** 1 Human-Machine Perception Laboratory, Department of Computer Science and Engineering, University of Nevada, Reno, Reno, NV, USA; 2 Michigan Medicine, University of Michigan, Ann Arbor, MI, USA; 3 Sidney Kimmel Medical College, Thomas Jefferson University, Philadelphia, PA, USA; 4 Brigham and Women's Hospital, Harvard Medical School, Boston, MA, USA; 5 University of Texas Health Science Center, Houston, TX, USA; 6 University College Dublin School of Medicine, Belfield, Dublin, Ireland; 7 Center for Space Medicine, Baylor College of Medicine, Houston, TX, USA; 8 Department of Ophthalmology, Blanton Eye Institute, Houston Methodist Hospital, Houston, TX, USA; 9 The Houston Methodist Research Institute, Houston Methodist Hospital, Houston, TX, USA; 10 Departments of Ophthalmology, Neurology and Neurosurgery, Weill Cornell Medicine, New York, NY, USA; 11 Department of Ophthalmology, University of Texas Medical Branch, Galveston, TX, USA; 12 University of Texas MD Anderson Cancer Center, Houston, TX, USA; 13 Texas A&M College of Medicine, TX, USA; 14 Department of Ophthalmology, The University of Iowa Hospitals and Clinics, Iowa City, IA, USA

**Keywords:** relative afferent pupillary defect, virtual reality, variability, extended reality, reliability

## Abstract

**Background:**

The swinging flashlight test (SFT) is one of the most prominent clinical tests for detecting the relative afferent pupillary defect (RAPD). A positive RAPD localizes the lesion to the affected afferent pupil pathway and is a critical part of any ophthalmic exam. Testing for an RAPD, however, can be challenging (especially when small), and there is significant intrarater and interrater variability.

**Methods:**

Prior studies have shown that the pupillometer can improve the detection and measurement of RAPD. In our previous research, we have demonstrated an automatic SFT by utilizing virtual reality (VR), named VR-SFT. We applied our methods to two different brands of VR headsets and achieved comparable results by using a metric, called RAPD score, for differentiating between patients with and without (control) RAPD. We also performed a second VR-SFT on 27 control participants to compare their scores with their first assessments and measure test–retest reliability of VR-SFT.

**Results:**

Even in the absence of any RAPD positive data, the intraclass correlation coefficient produces results between 0.44 and 0.83 that are considered of good to moderate reliability. The same results are echoed by the Bland–Altman plots, indicating low bias and high accuracy. The mean of the differences of measurements from test–retest ranges from 0.02 to 0.07 for different protocols and different devices.

**Conclusions:**

As variability among various VR devices is an important factor that clinicians should consider, we discuss the test–retest reliability of VR-SFT and the variability among various assessments and between two devices.

**Translational Relevance:**

Our study demonstrates the critical necessity of establishing test–retest reliability measures when bridging virtual reality technology into the clinical setting for relevant afferent pupillary defect.

## Introduction

The swinging flashlight test (SFT) is typically used to detect the relative afferent pupillary defect (RAPD). The SFT is quick, inexpensive, readily accessible, and easy to perform in clinic or at the bedside. The clinical manual SFT, however, has significant limitations, including the examiner's individual skill, the subjective nature of detection and quantification of the RAPD, and lack of standardization.^1^ Automation can reduce the inherent variability of the clinical SFT. Automated SFT can binocularly record pupillary light reflexes from light emitting diodes placed in front of each eye.^2^ By automating the SFT, measurements taken across institutions and practices may be more reproducible, stable, and reliable and can provide valuable quantitative measurements for following optic nerve disease and/or response to treatment.^2^

This may also improve applications of SFT as a more generalized screening method.^2^ Moreover, a subtle RAPD, when detected, may lead to early diagnosis of underlying disease.^3^^–^^5^ In addition to automation, virtual reality (VR) may play a key role in the next generation of SFT by producing a unique modality to run a preprogrammed and automated SFT. Commercial off-the-shelf VR headsets can make automated RAPD detection more accessible to a larger population. Incorporating a VR headset display of interactive virtual reality environments with incorporated visual tests for acuity, perimetry, and pupillary reflex can more objectively evaluate visual impairments.^3^ However, these emerging technologies must be validated and compared prior to clinical use. We have conducted experiments on a healthy population and measured their RAPD across two VR headsets, five (2 + 3) separate protocols, and two different sessions. In this article, our goal is to investigate four different aspects of our VR-based SFT.

1. Agreement between test–retest measurements by our systems (test–retest reliability)
2. Consistency between measurements of each subject by our systems (interrater consistency)
3. Agreement on RAPD quantification for all subjects for each system (intrarater agreement)
4. Similarity between our data and the data collected by earlier non-VR-based automated RAPD detection systems

Essentially, we want to understand whether our system rates a stable subject consistently across sessions, stimuli presentation (VR headset) hardware, and response (pupillary response) collection software. In the following sections, we will establish the related studies on RAPD detection, our approach to VR-based RAPD, and statistical analysis on understanding the reliability of different hardware–protocol pairing and discuss the implication of our findings for clinical use.

## Related Works

There are several causes of RAPD found in the literature ranging from infectious disease, vascular pathologies leading to optic disc pathologies, macular degeneration, glaucoma, and traumatic brain injury. RAPD is often found in the context of other ocular disorders. In patients with glaucoma, when compared to the control group, there was a statistically significant difference in pupil area ratio and pupil dilation velocity ratio showing how screening for RAPD may aid in the detection of neuropathy in glaucoma with high sensitivity and specificity.^4^ Folk et al.^5^ in 1987 observed that the degree of relative afferent pupillary defect can predict the extent of retinal detachment. Additionally, Kardon et al.^6^ studied static perimetry and RAPD and found that diseases that impact the afferent visual system may not necessarily affect visual threshold or pupillary light reflex in the same way, which created a key delineation in the diagnostic algorithm.

The use of SFT was the first of a long progression of innovations that have been worked on to detect RAPD. Thompson et al.^7^ in 1981 first reported on measuring RAPD, describing the methods and application of the SWT. In 1995, Kawasaki et al.^8^ described afferent asymmetry by running a computerized test that recorded pupillary response to alternating light stimulus. This was recorded using computerized infrared pupillography. Cohen et al.^9^ in 2015 validated their study on the use of novel computerized portable pupillometers for RAPD detection and observed a strong correlation between their pupillometer's results and expert examiner clinical grading suggesting pupillography as a novel clinical tool for RAPD diagnosis. In 2019, Temel et al.^10^ utilized transfer learning and introduced an automated framework to detect RAPD, reduce subjectivity, and improve test generalizability. Their three algorithms and nine performance metrics showed high levels of functionality and accuracy addressing several of the limiting factors of manual assessments like SFT for RAPD diagnosis and assessment.^10^ These advancements in machine learning may play a key role in the next-generation automation techniques developed for RAPD.

Research has been done to determine the variability of RAPD quantification using different illumination levels and shorter dark periods.^8^ The relationship between RAPD and many disorders, including glaucoma,^11^ optic nerve disease,^12^ and others, and the change in RAPD in patients with optic nerve diseases before and after treatment were also investigated by researchers.^13^ Additionally, studies have been done to determine the impact of gender and age on RAPD scores.^14^ The prevalence of RAPD in healthy persons was researched by Wilhelm et al.^15^ In this study, we aim to validate the reliability of VR-SFT by assessing two tests conducted over a short time period of 3 to 5 weeks. To accomplish this, we perform a reliability analysis of SFT undertaken in virtual reality and the data on pupil diameter obtained from it.

## Materials and Methods

### Subjects

Subjects for this research study were recruited from students and scholars of the University of Nevada, Reno. The research study was approved by the institutional review board. The inclusion criteria for the controls were age from 18 to 85 years and no history of RAPD. In total, 31 controls participated in the study. However, only 27 participants completed the study. The remaining participants only performed the first round of the study and were excluded from the evaluation of the variability. Out of the 27 participants, 8 participants were female, and 19 participants were male. The demographic details of the participants are described in Table 1. The history of ophthalmic pathologies in this cohort included refractive error, LASIK surgery, and cataract surgery. Written informed consent was obtained from the participants before their participation. In order to reduce the likelihood that results are the result of poor test stability rather than age-related changes in performance, test–retest reliability assessments were frequently taken over two time points during a brief period of time. In our case, the difference between two studies (test and retest) on the same participant ranged between 3 and 5 weeks.

**Table 1.** Participant Characteristics

Description	Total (Mean Age ± SD)	Male (Mean Age ± SD)	Female (Mean Age ± SD)
Number of participants	27 (27.33 ± 4.23)	19 (26.89 ± 3.65)	8 (28.38 ± 5.53)
Number of participants with history of ophthalmic pathology	20 (27.05 ± 4.76)	14 (26.71 ± 4.08)	6 (27.83 ± 6.46)
Number of participants without history of ophthalmic pathology	7 (28.14 ± 2.27)	5 (27.6 ± 2.30)	2 (29.5 ± 2.12)

### Devices and Experimental Software

For the research study, two different sets of VR headsets were used. The VR headsets used in the study were the HTC Vive Pro Eye (HTC Corporation, Xindian, New Taipei, Taiwan)^16^ and the FOVE 0 (FOVE Co., Ltd., Chiyoda-ku, Tokyo, Japan).^17^ The specifications of the headsets are given in Table 2. The programs for the VR environment were written in C++ programming language. Unreal Engine was used to integrate the programs with the VR headsets, which made the incorporation convenient and flexible. To perform the measurement of the pupil diameter, respective software development kit tools^18^^,^^19^ of the devices were used. The analysis was performed in Python programming language (version 3.9).^20^ The libraries used for calculation of intraclass correlation coefficient and Bland–Altman plot were pingouin (version 0.5.3)^21^ and pycompare (version 1.5.4).^22^

**Table 2.** Device Specifications of HTC Vive Pro and FOVE VR

Specification	HTC Vive Pro	FOVE VR
Resolution per eye	1440 × 1600	1280 × 1440
Maximum screen refresh rate, Hz	90	70
Field of view, degrees	110	100
Base station	Required	Not required

### Test Procedure

For our experiments, we followed the implementation method of VR-SFT.^23^ We used reduced light illumination to invoke artificial RAPD in the participants’ eyes. To eliminate the effects of accommodative response, visual stimuli were presented to make the illusion of the stimuli appearing from close to far distance. The participant's focus was on the visual stimuli during the whole procedure. There was a delay of 5 seconds after the visual stimuli winding up at far distance and the first light illumination. The delay was implemented so that the pupils could adjust to the dark adaptation.

**Figure 1.**
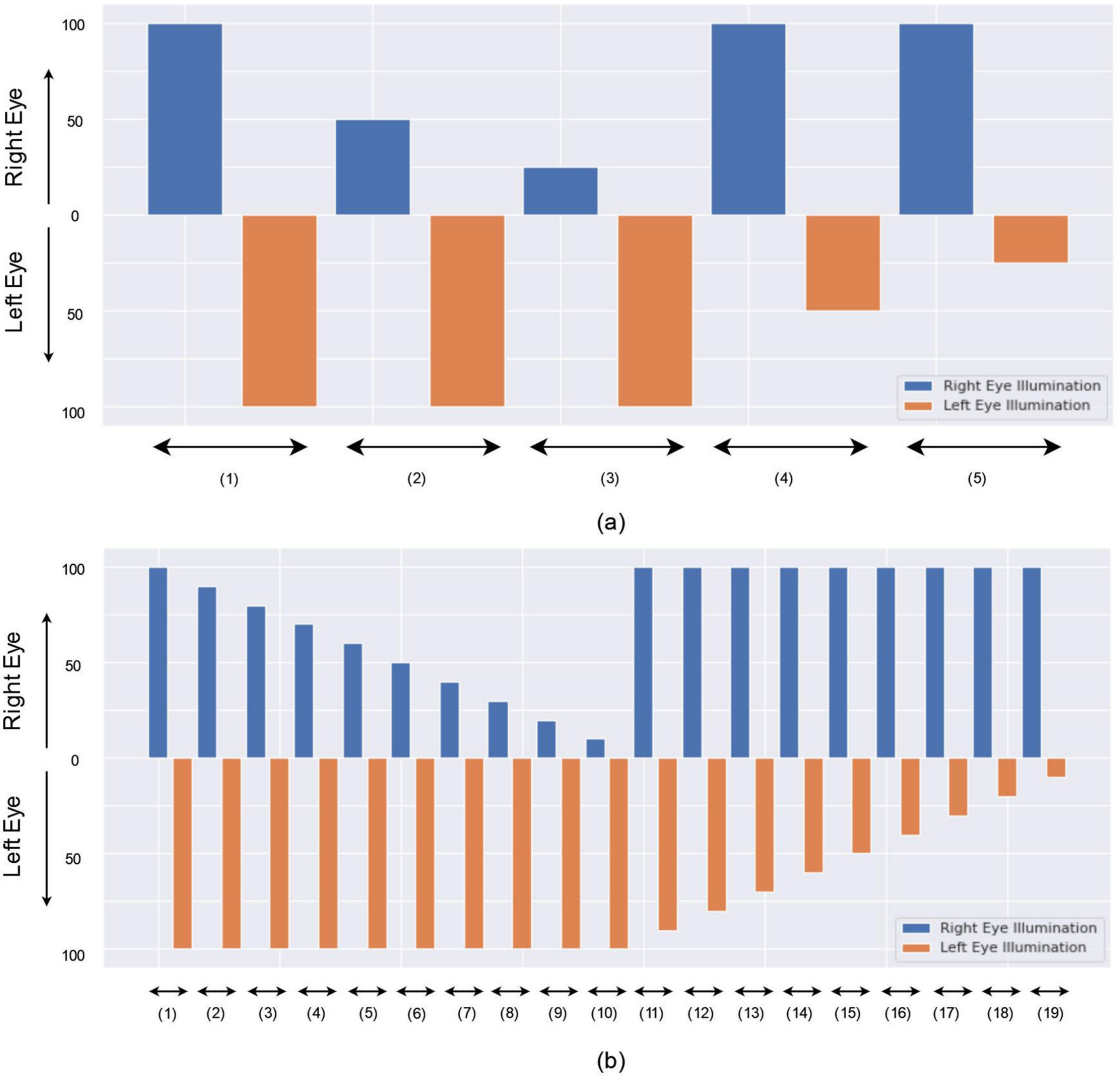
Plot for illumination level of (a) protocols 1 and 2 and (b) protocol 3. The x-axis represents the number of illumination levels. For each illumination level, respective right and left eye illumination is shown. For example, the second illumination of protocols 1 and 2 is 100% illumination on the left eye and 50% illumination on the right eye, which means at first, the right eye is illuminated with 50% of initial illumination before altering to the left eye with 100% of initial illumination. Each illumination level is repeated three times for protocols 1 and 2 and four times for protocol 3.

The tests were performed under different light intensity conditions to mirror the procedures of the RAPD testing with neutral density filters. We used three different protocols to measure the RAPD in controls. For protocols 1 and 2, we used 50% and 25% of the initial light intensity, which matched 0.3 and 0.6 log units for neutral density filters, respectively^23^ (Fig. 1). The initial light intensities for HTC Vive Pro and FOVE 0 VR devices were 97.8 and 97.2 cd/m^2^, respectively, which were similar for the traditional SFT.^15^^,^^20^ The light duration on each eye before altering to the other eye was 3 and 2 seconds for protocol 1 and protocol 2, respectively. Additionally, we devised a protocol 3 to get more data points for calculating the RAPD score as Kawasaki et al.^8^ discovered that the variability of a computerized determination of RAPD could be reduced by increasing the number of light stimuli and by using shorter dark periods between alternating light stimuli. In this protocol, we increased the intervals of light intensity with a step of 10%, and the light duration was kept to 2 seconds for this protocol. While protocol 1 and protocol 2 had 5 illumination levels, protocol 3 had 19 illumination levels because of the increased intervals (Fig. 1). For each illumination level in protocols 1 and 2, we repeated three times before moving to the next iteration, while in protocol 3, they were repeated four times. As the total duration of the protocol 3 was lengthy, we used 5 seconds of dark interval between each iteration to rest the eyes, and we only performed the experiments using the HTC Vive Pro device.^16^ The other two protocols were performed in both VR headsets. The total duration for protocols 1, 2, and 3 was 95, 65, and 400 seconds, respectively. More protocol details can be found in our previous work.^23^ Detailed specifications of the protocols are given in Table 3.

**Table 3.** Specifications of VR-SFT Protocols

Protocol Number	Light Intensity Levels in Each Eye, % of Initial Light Intensity	VR Devices Used	Light Duration on Each Eye, Seconds	Each Illumination Level Repetitions	Total Duration, Seconds
1	100, 50, 25	HTC Vive, FOVE 0	3	3	95
2	100, 50, 25	HTC Vive, FOVE 0	2	3	65
3	100, 90, 80, 70, 60, 50, 40, 30, 20, 10	HTC Vive	3	4	400

**Figure 2.**
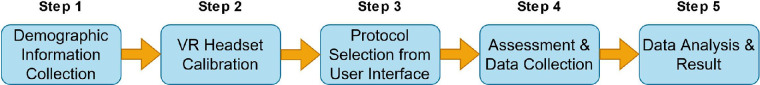
Summary of experiment procedure of VR-SFT.

After the tests were performed, the data were exported to a comma separated values (csv) file. The columns in the csv files were timestamps, light intensity in the right and left eyes, pupil diameters, and gaze locations for each eye. The data were kept in a secured folder in the cloud. The summary of steps of VR-SFT is shown in Figure 2.

### RAPD Quantification

**Figure 3.**
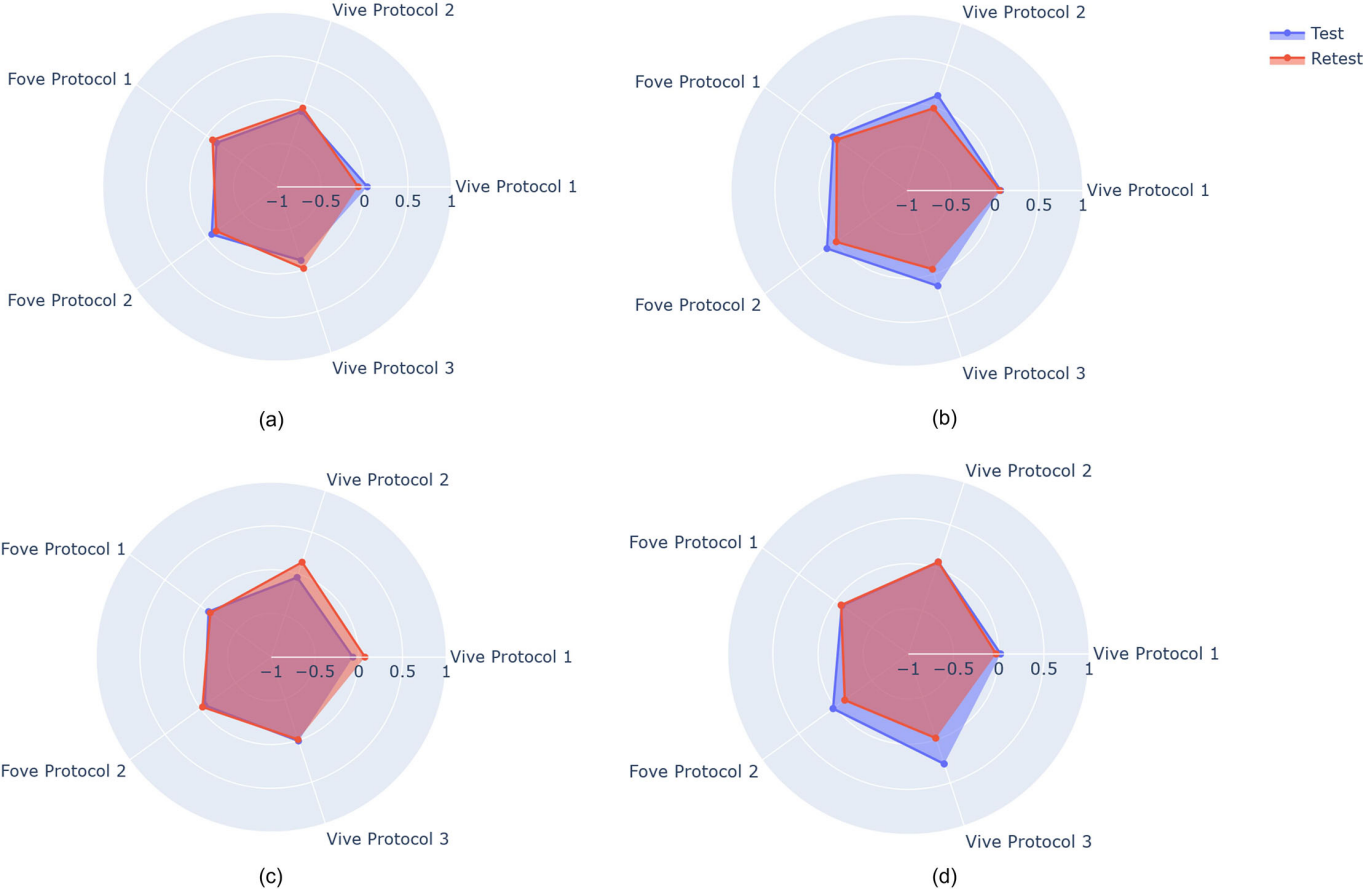
RAPD quantification of test and retest of four individuals under protocols 1, 2, and 3 for Vive and FOVE device. In most instances, test–retest evaluation results are extremely close.

Similar to the methods of Sarker et al.,^23^ we applied an RAPD score to quantify asymmetric pupillary responses in participants. Cohen et al.^9^ utilized the RAPD score with the RAPDx pupillometer (Konan Medical USA, Inc., Irvine, CA, USA). The authors demonstrated that the relationship of millimeter difference in contraction amplitude is linearly correlated with the difference of light illumination in log units. They plotted the individual pupil diameter difference on the y-axis with respect to illumination levels on the x-axis and used a regression line to locate the point where the line and x-axis cross. The intersection point is the RAPD score and determined by equal and balanced pupillary responses to stimulation of the right and left eyes created by log-unit attenuation of the stimuli. A positive intersection point represents RAPD in the right eye, while the negative intersection point represents RAPD in the left eye. The RAPD quantification from five distinct assessments of four patients who performed twice with a minimum 3-week interval is shown in Figure 3.

### Metrics

In our study, we used two correlation metrics, the intraclass correlation coefficient (ICC) and the Bland–Altman plot. In the ICC, the higher the reliability and the smaller the errors, the nearer R is to 1. The actual score variance and error variance are seen as contributing to the total variation in the data for a group of measurements. However, the difference in our case is that the true score of RAPD is unknown. So, the true score can be compensated for by using variance of subjects. Relative reliability in our case was calculated using the ICC with two-way mixed model for single measurement consistency and absolute agreement type reliability, respectively. To reiterate, correlation quantifies the relation between two or more different measurements of the same subject. However, high correlation is not sufficient to determine good agreement between the measurements. We used the Bland–Altman plot to determine agreement between quantitative measurements. The representation is a scatterplot with the x-axis representing the mean (t_1_ + t_2_)/2 and the y-axis representing the difference (t_1_ – t_2_) of measurements.

## Results

**Figure 4.**
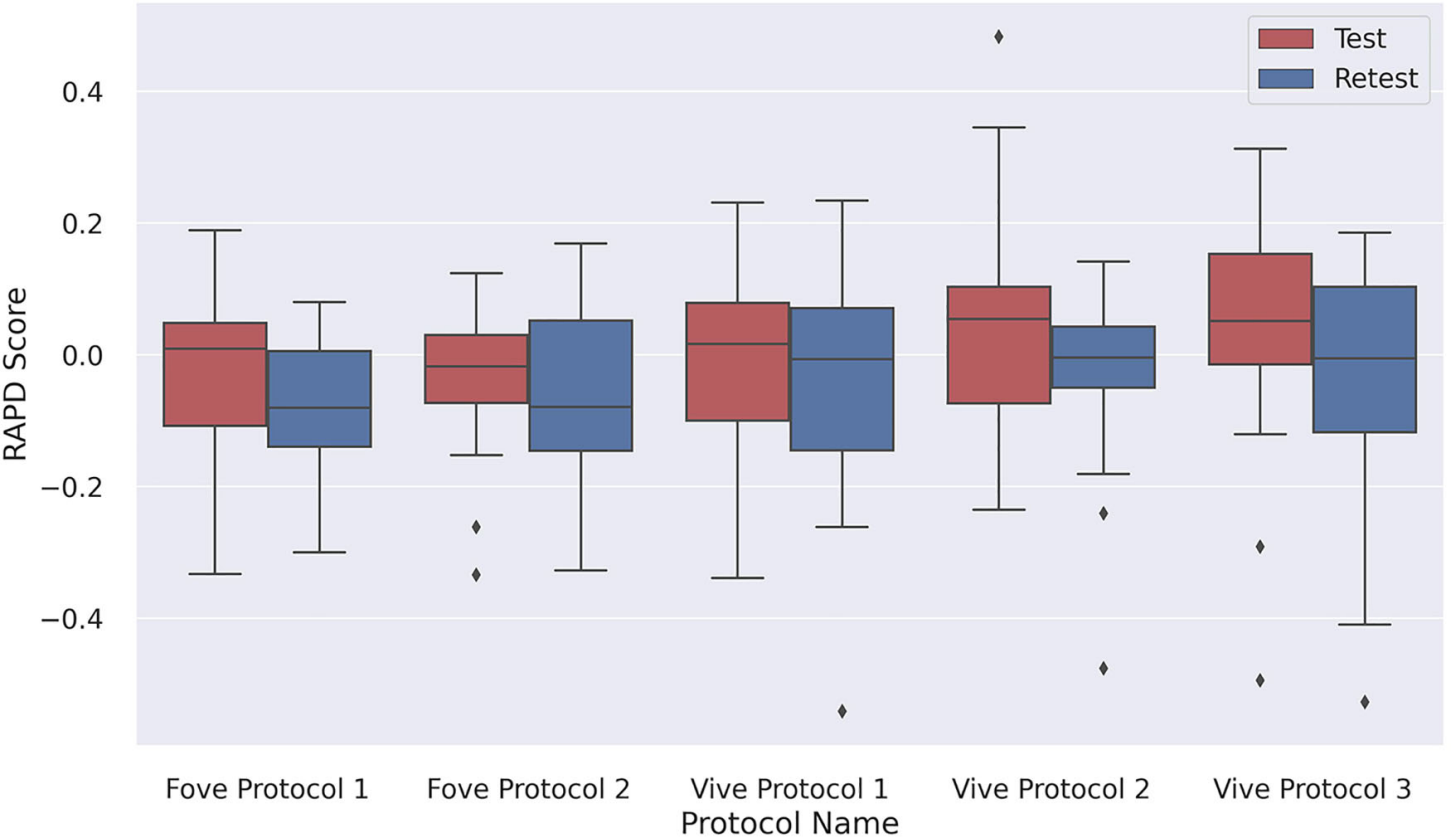
Boxplot showing distribution of RAPD score of each device and protocol for test and retest.

The boxplot of the distribution of RAPD scores for each setting and test and retest values are shown in Figure 4. The boxplots demonstrate a visual summary of the data, including mean values, the dispersion of the data set, and the higher and lower limits with most of the boxplot results between the normal range of –0.3 and 0.3. The outliers of the boxplots are anomalies of the control group.

In Table 4, we demonstrate our analysis of the variability between test and retest of individuals from control group. For this analysis, a two-way mixed model for single measurement consistency was used. Reliability comparisons between devices and protocols are shown in Tables 5 and 6. The two-way mixed model for single measurement is shown to demonstrate absolute agreement reliability.

**Table 4.** Test–Retest Variability

Protocol	Number of Participants	ICC	95% Confidence Interval	*P* Value
FOVE protocol 1	20	0.83	0.48–0.95	4 × 10^−4^
FOVE protocol 2	20	0.80	0.41–0.94	9 × 10^−4^
Vive protocol 1	27	0.53	0.15–0.77	4 × 10^−3^
Vive protocol 2	27	0.44	0.04–0.72	2 × 10^−2^
Vive protocol 3	26	0.57	0.21–0.8	2 × 10^−3^

**Table 5.** Variability Between Devices

Protocol	Number of Participants	ICC	95% Confidence Interval	*P* Value
FOVE protocol 1 and Vive protocol 1	20	0.46	0.15–0.69	3 × 10^−3^
FOVE protocol 2 and Vive protocol 2	20	0.64	0.38–0.81	1 × 10^−5^
FOVE protocol 2 and Vive protocol 1	20	0.52	0.23–0.73	7 × 10^−4^
FOVE protocol 1 and Vive protocol 2	20	0.53	0.24–0.73	5 × 10^−4^
FOVE protocol 1 and Vive protocol 3	20	0.58	0.29–0.77	3 × 10^−5^
FOVE protocol 2 and Vive protocol 3	20	0.56	0.27–0.76	2 × 10^−4^

**Table 6.** Variability of Results From Same Devices and Different Protocols

Protocol	Number of Participants	ICC	95% Confidence Interval	*P* Value
FOVE protocol 1 and FOVE protocol 2	20	0.82	0.67–0.91	3 × 10^−9^
Vive protocol 1 and Vive protocol 2	27	0.62	0.42–0.76	5 × 10^−7^
Vive protocol 1 and Vive protocol 3	26	0.66	0.46–0.79	1 × 10^−7^
Vive protocol 2 and Vive protocol 3	26	0.75	0.58–0.85	1 × 10^−9^

We performed our testing in the HTC Vive device first and then with FOVE 0. In total, 27 participants completed protocols 1 and 2 of the study with the HTC Vive device. Out of them, 26 participants finished the protocol 3 assessment with the HTC Vive device. Some participants did not complete the test or retest with FOVE 0 because they did not want to continue the test because of the strain on the eyes from the earlier testing or blinked too much during the test, making the data inapplicable. Therefore, the number of participants was less for assessments with FOVE. 20 control participants completed protocols 1 and 2 of the study with FOVE 0.

**Figure 5.**
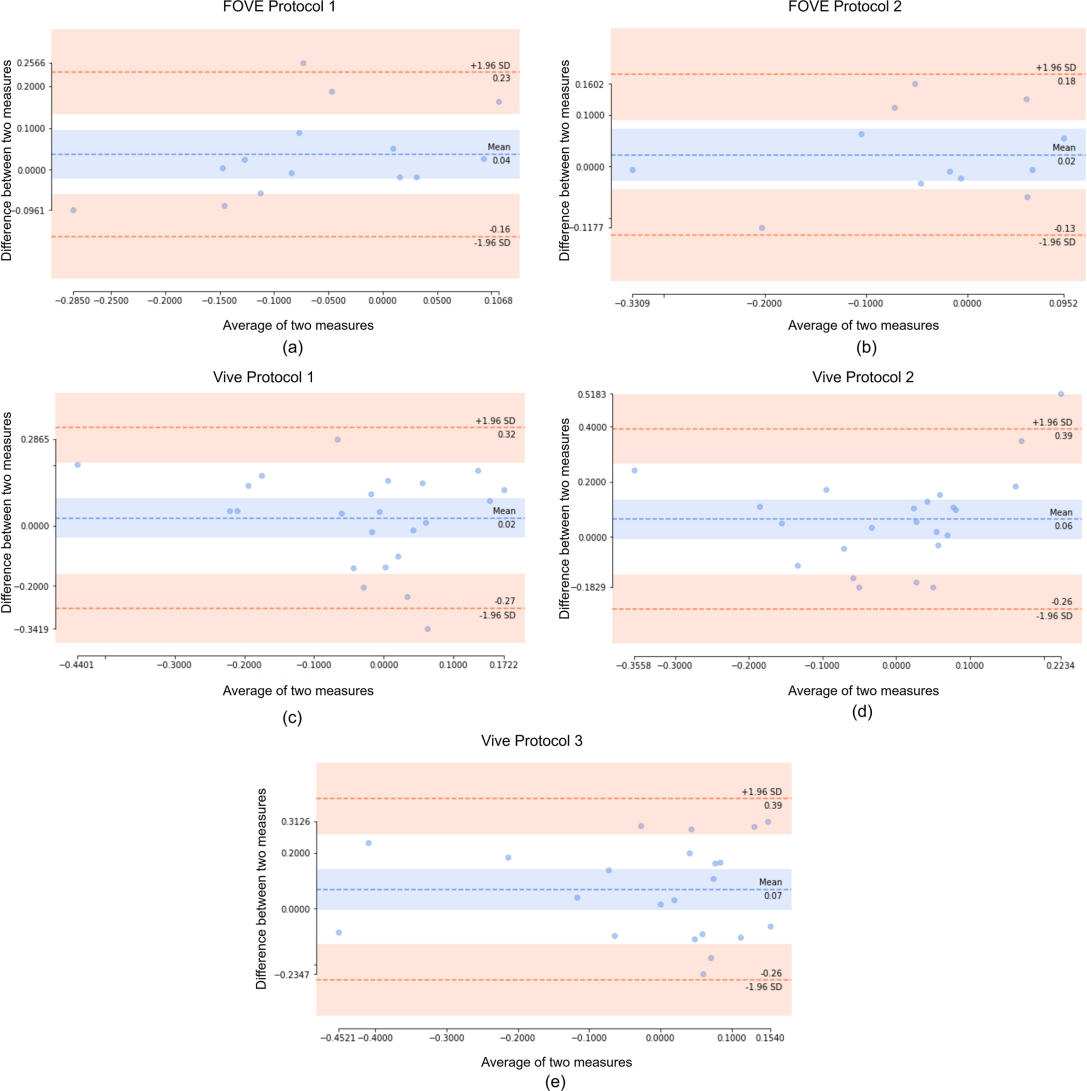
Bland–Altman plot of each device and protocol.

Figure 5 shows five different Bland–Altman plots demonstrating the mean of test and retest RAPD scores on the x-axis and the difference between test and retest on the y-axis. The central horizontal line is the mean of the differences. The closer the data points are to the line of identity, the more agreeable the test and retest measurements. This aspect represents the accuracy of the system. The top and bottom dashed lines represent the limits of agreement (LoA), where LoA is 1.96 * SD (standard deviation) of differences. When most of the data lie within this range, it signifies the precision of the system. The blue area at the top and bottom of the mean value represents the 95% confidence interval of the scores.

## Discussion

In this study, we report test–retest variability of results from the VR-SFT. We also present variability of results between two devices and three different protocols. For our analysis, we chose to employ ICC and the Bland–Altman plot. The interpretation of Cicchetti^24^ regarding the ICC values is as follows: poor reliability can be determined if ICC is less than 0.4, fair reliability if the ICC value is between 0.4 and 0.59, good reliability if the ICC is between 0.6 and 0.74, and excellent reliability if the ICC is between 0.75 and 1. There is also a recent interpretation of Koo and Li^25^ that describes an ICC value of 0.5 as “poor,” ICC value between 0.5 and 0.75 as “moderate,” ICC value between 0.75 and 0.9 as “good,” and ICC value above 0.9 as “excellent.”

In our analysis, test–retest variability of the same device and protocol using two metrics, ICC (Table 4) and Bland–Altman plot (Fig. 5), demonstrated agreement between results from different devices and protocols using ICC (see Tables 5 and 6). For test–retest reliability, the highest ICC value was 0.83 for FOVE protocol 1, and the lowest ICC value was 0.44 for the Vive protocol 2. We can notice that ICC values for FOVE protocol 2 and Vive protocol 2 were lower than their respective protocol 1. This indicates that protocol 1 with a 3-second light duration was more reliable in producing consistent scores than protocol 2 with a 2-second light duration. The high ICC score of FOVE protocol 1 and protocol 2 indicates any test performed on this device and protocol shows a relatively high reliability result. ICC scores with the Vive devices are in the range of moderate and fair reliability.

To show the reliability among different protocols and devices, we calculated ICC (Tables 5 and 6). The reliability was higher when the same device was used for different protocols. In those cases, the ICC yielded in the good and moderate range. Variability between devices was slightly lower. However, the scores remained in the fair and moderate range.

An important aspect of our analysis is that all RAPD scores came from control data, and the distribution's SD would have been higher if it also had included RAPD-positive patient data. Low ICC could be related to the homogeneity of the sampled patients.^25^ The ICC scores were moderately high despite the distribution's lack of variation. This demonstrates the capability of our method to yield comparable results. Again, the 95% confidence interval illustrates that there is a 95% likelihood that the true ICC values will fall somewhere between the lower and upper limits of the interval.^25^ In our case, the upper bound of the interval was higher when the ICC score was higher. If the distribution contained more data points and had greater heterogeneity, the difference of the range would have been lower. Additionally, in every test–retest, intradevice, and interdevice reliability situation, the *P* value was consistently much lower than 0.05, indicating the statistical significance of our methodologies.

When compared with the ICC values for test and retest, we can see that the Bland–Altman plots reiterated the same properties (Fig. 5). FOVE protocols 1 and 2 both showed considerably smaller difference values than the Vive protocols, which was reflected in higher ICC values. Even though FOVE protocol 2 and Vive protocol 1 showed similar bias values, the spread was much greater in Vive protocol 1. Crucially, for FOVE, most samples were within the 95% confidence interval for RAPD test–retest score difference. For all the test–retest devices and protocols, the line of identity was within the confidence interval, signifying low bias and high accuracy. Some points in Vive protocols showed a high difference between test and retest, which may have led to completely different diagnostic conclusions for physicians. However, in the Vive protocols, the extreme scores (|RAPD|>0.3) had a relatively small test–retest difference, thereby mitigating the chance of wrong classification. Importantly, we can see that for most of the samples, the test and retest scores had a mean within the normal ranges, as expected. For examples that were outside the normal ranges (−0.3, 0.3), the FOVE protocols appeared to be more consistent than the Vive protocols.

The findings of our analysis are consistent with those reported in earlier literature in terms of the distribution of RAPD scores in controls. For instance, Satou et al.^14^ and Wilhelm et al.^15^ discovered that the distribution of RAPD scores in people with normal vision lies between –0.5 and 0.5 as it is estimated that an RAPD may be detected with these techniques in up to 13% of the population with normal vision.^15^ Except for one subject with a history of prior refractive surgery, we also discovered the same distribution of RAPD scores. The subject's RAPD score in the second round of the study was determined to be greater than 0.45 log units for all protocols.

## Conclusion and Future Works

Our study found significant variability in the reliability and reproducibility of RAPD scores using various protocols and VR devices, but standardized scoring indices and measurement protocols improved objective results. The correlation coefficients ranged between moderate and good even in the absence of heterogeneity of the data. In the next generation of automated RAPD measurement, machine learning algorithms integrated with virtual reality modalities will likely further improve the accessibility and generalizability of the technology for clinical use.

For our future work, we intend to examine the validity of our approach on both controls and participants who tested positive for RAPD in order to increase the heterogeneity of the data. Although there are other pupil variables to consider, in our study, we solely evaluated pupil diameter amplitude to diagnose RAPD. We are attempting to develop a metric that includes these variables as part of our ongoing research.

Our study has the potential to have substantial translational value since it can give clinicians a reliable and objective tool to track the development of the disease and the effectiveness of treatment over time. This may be particularly useful in clinical studies examining novel therapies for neurological diseases that affect the visual system. This will make it easier to translate cutting-edge discoveries and technologies into efficient patient diagnostic and therapeutic approaches. Additionally, the use of virtual reality technologies for RAPD evaluation can make the diagnostic procedure more interactive and engaging, resulting in better patient compliance and early identification and treatment of visual dysfunction, which can enhance patient outcomes. The accessibility and generalizability of the technology for clinical usage can be further enhanced by standardized scoring indices and measurement methodologies, as well as the continued development of machine learning algorithms linked with virtual reality modalities.

Variability among VR devices remains a critical potential limitation of bringing automation of the assessment of the RAPD into clinical practice. The emergence of VR-based testing for clinical care remains promising, but further work is necessary as the technology continues to evolve to improve reproducibility, reliability, and validity. Following those steps, the test–retest reliability of VR devices can provide a reliable and objective tool for assessing neurological conditions that affect the visual system, bridging the gap between basic research and clinical care.
